# Using Computational Neuroscience to Define Common Input to Spinal Motor Neurons

**DOI:** 10.3389/fnhum.2016.00313

**Published:** 2016-06-21

**Authors:** Tjeerd W. Boonstra, Simon F. Farmer, Michael Breakspear

**Affiliations:** ^1^Black Dog Institute, University of New South WalesSydney, NSW, Australia; ^2^Systems Neuroscience Group, QIMR Berghofer Medical Research InstituteBrisbane, QLD, Australia; ^3^Sobell Department of Motor Neuroscience and Movement Disorders, University College LondonLondon, UK; ^4^National Hospital for Neurology and NeurosurgeryLondon, UK; ^5^Metro North Mental Health Service, Royal Brisbane and Women's HospitalBrisbane, QLD, Australia

**Keywords:** common input, motor neurons, connectivity analysis, computational modeling, synchronized oscillations, coherence analysis, spiking neurons

Common input is a widely used concept in motor neurophysiology. It embodies the notion that inputs to individual spinal motor neurons (MNs) are not unique, but partly shared across MNs, and is considered the main explanation for synchronized activity of MNs (Bremner et al., 1991; Farmer et al., 1993; Boonstra and Breakspear, 2012; Farina and Negro, 2015). Motor-unit synchronization was first observed in the time domain using cross-correlation histograms from pairs of MNs (Sears and Stagg, 1976). This was later extended to the frequency domain by estimating coherence between spike trains to reveal the frequency content of common input (Farmer et al., 1993). In addition to measurements of individual MNs, coherence can also be estimated between the surface EMG of different muscles—referred to as intermuscular coherence—to assess common input shared across motor-unit pools (Boonstra and Breakspear, 2012). Common input is considered relevant for motor control as it may provide a mechanism to reduce the dimensionality of the control signal, thereby simplifying motor control (Farmer, 1998).

Recently the existence of common input has been debated: while some argue that synergistic muscles share most of their synaptic input (Laine et al., 2015), others have argued that common input provides no explanation for MN synchronization (Kline and De Luca, 2016). Apart from common input being used to explain different types of MN synchronization, this dispute mainly arises from the absence of a clear definition of common input (cf. Kirkwood, 2016). Initially, Sears and colleagues defined common input structurally, that is, as resulting from branched presynaptic axons (Sears and Stagg, 1976; Kirkwood and Sears, 1978). Later, presynaptic synchronization—synchronization of neurons that project to the MNs—was proposed as an explanation for broad-peak MN synchronization (central peak in cross-correlogram with a duration of 40–60 ms), which is observed following a lesion of the direct mono-synaptic inputs to MNs and cannot be explained by branched presynaptic axons (Kirkwood et al., 1982). This is a functional definition as it involves correlations between input activities, rather than shared anatomical connections. Although the authors themselves did not refer to this mechanism as common input, the term common input has been invoked quite loosely in recent years, reflecting either shared structural or functional inputs.

To resolve this dispute and determine whether MN synchronization is caused by common input, we first need to agree on the definitions of common input and MN synchronization. Computational models are particularly useful as they provide a quantitative and unambiguous description of variables. Computational neuroscience can be used to define common input in terms of a set of equations and determine its effect on MN synchronization (Boonstra, 2013).

We therefore applaud the contributions by Farina and colleagues who provided equations to define common input (Castronovo et al., 2015; Farina and Negro, 2015). They describe the inputs to MNs as

(1) $$\begin{array}{r} v_{i}\left(t\right)=\mu_{i}\;+\;\alpha_{i}s^{C}\left(t\right)+n_{i}\left(t\right), \end{array}$$

where *v*_*i*_(*t*) is the synaptic current to the *i*-th MN, μ_*i*_ a constant offset, $\alpha_{i}s^{C}$(*t*) the time-varying input that is common to all MNs (apart from a constant scaling factor α_*i*_), and *n*_*i*_(*t*) is independent white noise. The distinction between μ_*i*,_ $\alpha_{i}s^{C}$(*t*) and *n*_*i*_(*t*) is not based on anatomical constraints, i.e., where these inputs originate from, but rather decomposes the total input signal into a DC, a correlated and an uncorrelated component, respectively. This is therefore a functional definition, as common input is defined in terms of correlations between input activities.

Using this formal definition, the effects of common input on force production and MN synchronization can be investigated using analytic and computational approaches. Farina and Negro (2015) show that, when defined in this way, common input is the only input component that influences force generation and that correlated activity of MNs is thus necessary for force control. This is a consequence of the approximately linear input-output relationship of a MN pool (Stegeman et al., 2010; Farina et al., 2014). To generate force muscles require a net excitatory drive and hence the cumulative effects of correlated inputs. This is because uncorrelated inputs average out as they consist of numerous excitatory and inhibitory inputs (Farina et al., 2014; Farina and Negro, 2015). Using this functional definition the notion that MN synchronization is caused by common input becomes therefore true by necessity. Indeed, a coupling between oscillators is necessary for synchronization to occur (Pikovsky et al., 2001). That is, if the outputs of MNs are correlated, the inputs of MNs need to be correlated (functional definition of common input). However, correlated inputs do not necessarily mean these inputs originated from a common source (structural definition of common input).

While a positive step toward standardization, the proposed definition of common input risks tautology and it does not address the underlying neurophysiological cause of correlated inputs. We propose an alternative definition of common input consistent with its original interpretation (cf. Perkel et al., 1967), one that builds on a well-established approach in computational neuroscience, and that is to introduce structural constraints in the definition of common input. Thus we will use a formal biophysical modeling framework and consider a more realistic connection topology. First, we recast Equation (1) within the framework of a spiking MN,

(2) $$\begin{array}{r} C\frac{dV_{i}}{dt}=-g_{i}\left(V_{i}-E_{i}\right)+v_{i}\left(t\right), \end{array}$$

where *V*_*i*_ is the membrane potential of the *i*-th MN (Boonstra and Breakspear, 2012; Heitmann et al., 2015). Note here that the total synaptic current *v*_*i*_(*t*) from the LHS of Equation (1) appears here as the second term on the RHS. When this synaptic current is sufficient to push the membrane potential past a threshold (*V*_*T*,_ the spiking threshold), the neuron fires a spike and is instantaneously reset to a lower value (*V*_*R*,_ the reset potential). This is the simplest (integrate and fire) spiking model—and an abstract model of MNs (see Heckman and Enoka, 2012, for a review of MN physiology)—with a single leaky current given by the first term on the RHS with Nernst potential *E* and conductance *g* (Burkitt, 2006). If the summed synaptic currents *v*_*i*_(*t*) exceed the leaky current, the membrane potential rises toward the firing threshold *V* = *V*_*T*_ whereupon the neuron issues a single spike and is reset to its resting potential: this effect of spiking and resetting essentially incorporates the first order influence of all voltage-dependent channels in more complex models such as the Hodgkin-Huxley model (Kistler et al., 1997).

The next step is to recognize that the total synaptic current *v*_*i*_(*t*) into the cell soma is a filtered version of all small and transient currents arising at synapses following presynaptic spikes:

(3) $$\begin{array}{r} C\frac{dV_{i}}{dt}=-g_{i}\left(V_{i}-E_{i}\right)+\alpha \sum_{j}c_{ij}F\left(t,t_{j}\right), \end{array}$$

where *F* is a function that represents synapto-dendritic filtering and turns the discrete presynaptic spike times into smooth currents within the soma; *c*_*ij*_ represent the effective influence by presynaptic neurons *j* on the post-synaptic MN *i* (Gerstein et al., 1989), and α is a scaling function that modulates the total sum of all synaptic currents from the dendritic tree into the soma. Negative weights in *c*_*ij*_ can be used to model inhibitory inputs. For each MN *i, c*_*ij*_ is a vector weighting all possible sources *j* projecting to the MN. Presynaptic spikes for each neuron are recorded in the spike train *t*_*j*_ and usually only the most recent spike time is recorded, i.e., when a new spike arrives, this is reset to *t*_*j*_ = *t*. The dendritic filter can take several forms, but usually a simple form that allows exponential rise and decay is used,

F(t,tj)=e(tj−tτ1)−e(tj−tτ2)

where τ_1_ is the rise time and τ_2_ is the decay time (Kirkwood and Sears, 1978; Burkitt, 2006).

Finally, as with Equation (1), it is usual to introduce a noisy (stochastic) term to acknowledge that there are membrane fluctuations that do not solely reflect input spike trains: these may be due to weak synaptic currents not explicitly modeled as presynaptic spikes, or from truly random events such as the inherently noisy opening and closing of membrane channels (Faisal et al., 2008). Hence we have

(4) $$\begin{array}{r} C\frac{dV_{i}}{dt}=-g_{i}\left(V_{i}-E_{i}\right)\;+\;\alpha \sum_{j}c_{ij}F\left(t,t_{j}\right)\;+\;\eta_{i}\left(t\right), \end{array}$$

where η_*i*_(*t*) is (zero mean) white or colored noise, unique to each time step and neuron.

What are the differences between Equations (1) and (4)? First, the influence is modeled not just at the level of synaptic inputs but rather at the cell membrane of MNs, which are the ultimate effectors (through their own spikes) on muscle activity. The more complex formulation of Equation (4) allows the variable influence of spikes on the cell membrane and the balance between incoming and leaky currents. Hence the result is not a passive algebraic addition as in Equation (1), but rather a time dependent process that filters and integrates the weighted synaptic inputs.

Second, a generic coupling matrix *C* = {*c*_*ij*_} allows one to model any potential configuration of inputs, and should be constrained by knowledge of anatomy. The input configuration in Equation (1) can be obtained by considering a single source *j*_1_ that projects to all motoneurons *i*, as reflected by strong weights along the corresponding row of the coupling matrix. Each neuron also has an independent stochastic term η_*i*_ and the amount of common input can hence by varied by changing the scaling function α. Comparing Equation (1) and Equation (4), it hence seems that—rather than defining the ratio of common and individual MN input—the model by Farina and colleagues (Castronovo et al., 2015; Farina and Negro, 2015) defines the ratio between input and noise, as the noise term η_*i*_ captures weak currents that do not reflect input spike trains.

Equation (4) is very generic and the inputs *t*_*j*_ can be temporally homogeneous or oscillatory and uncorrelated or synchronized. Likewise, the coupling matrix C can be extended to include multiple input sources arising from descending and ascending pathways and spinal interneurons (Latash, 2008), thus enabling a more realistic connection topology. Realistic coupling matrices may be high dimensional. To reduce dimensionality and make the system more tractable, a mean-field approximation can be used to consider connectivity between ensembles of neurons (like a motor neuron pool) rather than between individual neurons (Deco et al., 2008). When estimating connectivity from empirical data, standard dimension reduction approaches can be used to obtain a more low dimensional representation (e.g., Roca et al., 2009).

Using Equation (4), independent inputs reflect presynaptic neurons that selectively innervate a single MN, as reflected by a strong connection weight and zeros along the rest of the corresponding row of coupling matrix *C*. In contrast, common inputs are reflected by strong connection weights along the whole row *j*. The amount of common input between two MNs can thus be quantified by the correlation between the weights in the two corresponding columns *i*_1_ and *i*_2_ of *c*_*ij*_. This definition of common input is a structural definition of common input, as it quantifies the proportion of anatomical connections shared between MNs. The difference between the functional and structural definition of common input can be illustrated by a simple example: consider a presynaptic neuron *j1* innervates MN *i1* and a presynaptic neuron *j2* innervates MN *i2*. If the spike trains of *j1* and *j2* are correlated, this would be considered common input according to Equation (1). In contrast, this is not considered as common input according to Equation (4), as the rows of *c*_*ij*_ are not correlated.

We hence follow the definition by Sears and Stagg (1976), but extend it to include all structural connections that are shared across MNs. A key attribute of Equation (4) is that it links structure and function, that is, it relates functional connectivity (observed MN synchronization captured by *V*_*i*_) to effective connectivity (captured by coupling matrix *c*_*ij*_). Effective connectivity depends on some model of the influence one neuronal system exerts over another (Friston, 1994; Horwitz, 2003). By providing a structural definition of common input in Equation (4), we position the debate on common input within the broader context of structure-function relationship in computational neuroscience (Honey et al., 2007; Bullmore and Sporns, 2009).

Having a realistic generative model may have important advantages. In particular, MN firing patterns can be “reverse engineered” to identify the underlying organization of synaptic inputs and intrinsic properties of MNs (Heckman and Enoka, 2012). For example, a Bayesian framework can be used to estimate effective connectivity from observed functional connectivity (Friston et al., 2003). We recently showed that functional connectivity between leg muscles reveals a rich structure of connections between muscles (Boonstra et al., 2015). Using model inversion techniques these functional connectivity patterns may be used to uncover structural pathways in the motor system that are difficult to assess directly.

In summary, we propose a structural definition of common input by using standard, well-established models in computational neuroscience. Rather than defining common input as the correlation between presynaptic activities, we suggest to define common input as the correlation between the anatomical connections or structural links that innervate MNs. A structural definition of common input steers the debate toward a pertinent research question: What are the neural pathways that cause the different types of synchronization that are experimentally observed between pairs and groups of MNs?

## Author contributions

TB, SF, and MB contributed to the conception and design of the work. TB drafted the initial version of manuscript. TB, SF, and MB revised the manuscript and approved the final version.

### Conflict of interest statement

The authors declare that the research was conducted in the absence of any commercial or financial relationships that could be construed as a potential conflict of interest.
